# Mechanical and electronic properties of van der Waals layered hcp PdH_2_

**DOI:** 10.1038/s41598-020-61385-5

**Published:** 2020-05-15

**Authors:** Zeliang Liu, Rajeev Ahuja, Huijian Li, Wei Luo

**Affiliations:** 1 College of Civil Engineering and Mechanics, Yanshan University, Qin Huangdao, Hebei, 066004 China; 2 Materials Theory Division, Department of Physics and Astronomy, Uppsala University, Uppsala, S75121 Sweden; 3 Department of Materials and Engineering, Applied Materials Physics, Royal Institute of Technology (KTH), SE-100 44 Stockholm, Sweden

**Keywords:** Materials science, Physics

## Abstract

Mechanical and electronic properties of palladium dihydrides (PdH_2_) as a function of pressure were studied by ab initio calculations based on density functional theory (DFT). The ab initio random structure searching technique was employed for screening potential PdH_2_ crystal structures under high pressure. A hexagonal close packed (hcp) phase of PdH_2_ with space group P6_3_mc was reported. The structure geometry and elastic constants were calculated as a function of pressure. It was found that H atoms are in the interstitial position of Pd atoms layer at 0 GPa. There is an electronic topology transition of hcp PdH_2_ at 15 GPa. When pressure exceeds above 15 GPa, one hydrogen atom occupies the tetrahedral site and another hydrogen atom locates in the interstitial position. When the c/a ratio is between 1.765 to 1.875, the hcp PdH_2_ is mechanically stable, and the Pd-H_2*b*_ bond is the major factor that limits the mechanical stability. The elastic constant C_44_ is the first one that cannot satisfy the mechanical stability criteria under pressure. The anisotropy parameters are far from 1(one) shows that the hcp PdH_2_ is a highly anisotropic structure. The electronic structure study indicates that the bonding force between Pd and H atoms along the z-axis direction increases with the increasing pressure. Also, the phonon dispersion study shows that PdH_2_ is dynamic stability under pressure. The results suggest that hcp PdH_2_ can be metastable in van der Waals layered structure.

## Introduction

Metal hydride (M-H) systems have attracted a lot of attention because of their properties, such as high hydrogen-storage capacity, fast hydrogen absorption/desorption, long-term cycle life and low toxicity^1^. Palladium-hydrogen (Pd-H) is used to be a system to understand the hydrogen atoms bonding with the metal host lattice in the M-H systems. The phase diagram and electronic properties of Pd-H have been used as a prototype in other M-H systems^2^. Some applications of Pd-H system have been investigated. The palladium can absorb hydrogen at ambient conditions and the dissociative adsorption of H_2_ molecules occurs with little or no activation energy barrier on the palladium surface. The reversible hydrogen absorption property can be used for hydrogen storage^3^. Increasing the hydrogen concentration will cause volume expansion of PdH_*x*_. Based on this mechanism, a Pd based H_2_ sensor was used to measure the hydrogen concentration^4^. Due to the dissociative properties of H, Pd-H can be used for hydrogen-related catalytic reactions^5^.

The hydrogen concentration affects the superconductivity of Pd-H system. PdH_*x*_ is a superconductor and the transition temperature Tc increased with the increase of H concentration x^6^. For the H/Pd is 0.81, a Tc $=$ 1.3 K was observed. At the highest concentration ratio of about 1.0, the Tc to the superconducting state higher than 8.0 K^7^. Recently Syed *et al.* found that by rapidly cooling the hydride after loading with hydrogen at elevated temperature, the Tc has a remarkable increase to 54 K when the H/Pd is about 1^8^. Tripodi *et al*.^9^ have shown that, when the H/Pd is up to 1.6, the Tc can go up to room temperature. Hence, the PdH_2_ could be considered as a possible room temperature superconductor.

The hydrogen concentration also affects the characteristics of the Pd-H system including mechanical, hydrogen storage capability and superconductivity^10^. The hydrogen storage capacity of the Pd-H system increases with the increase of H concentration. For the stoichiometry PdH, palladium can absorb 935 times its volume in hydrogen gas^11^. Neutron diffraction study has been performed for PdH_*x*_ system. The maximum hydrogen concentration is 0.7 at room temperature and normal pressures^12^. It was found that the hydrogen reduces the compression resistance in Pd-H^13^, in which bulk modulus decreases from 195 GPa to 183 GPa with the increase of H concentration from 0 to 0.76 indicating. It has been reported by Greenwood and Earnshaw that the metallic conductivity reduces as hydrogen is absorbed of PdH_*x*_ reduces with the increase of x, until at around PdH_0.5_ the solid becomes a semiconductor^14^. The H concentration x in PdH_*x*_ depends on the temperature and pressure. By heating the Pd film to 600 °C in a hydrogen atmosphere at pressure 0.1 GPa, a high H concentration phase PdH_1.33_ was obtained^15^. PdH_2_ has also attracted the attention of researchers because of the increase of H concentration compared with monohydride^16^. Experiments show that PdH_*x*_ can be formed on the top of multi-wall carbon nanotubes^17^. For 0 < x < 1, it is stable in face-centered cubic (fcc) structure. While, for x = 2 it has a hexagonal close-packed (hcp) structure. Palladium was studied in a high pressure hydrogen atmosphere up to 20 GPa. However, the PdH_2_ was not observed by X-ray diffraction until the pressure up to 20 GPa^18^.

Ab initio studies have been performed for the stability of PdH_2_ structure. It was found that the PdH_2_ is octahedrally-centered and H_2_ dimer is inside the fcc PdH_2_. The H_2_ dimer and octahedron have the same axis. There is repulsive interaction between the H atoms in the octahedral cage, which causes two atoms move to the tetrahedral sites^19^. The chemical bonding properties were studied by analyzing the electronic structure and partial density of states (PDOS). The overlap of Pd 4d-H 1s states is the most important for stabilizing the dihydride. However, the antibonding Pd 4d-H 1s states set in lower energy because of the downshift of the central group of 4d bands. As a consequence, the destabilization of dihydride was observed as compared to PdH^20^. The formation enthalpy calculations show that the PdH_2_ in fcc structure is unstable and this will decompose into fcc PdH and H_2_^21,22^.

In the present work, the random search study was performed to predict the structure of PdH_2_ at ambient as well as at the high pressures. An energy minimum structure is confirmed. It is an hcp structure, and the space group is P6_3_mc. The structural parameters of hcp and fcc PdH_2_ are discussed as a function of pressure. The elastic constants of hcp PdH_2_ as a function of pressure are obtained through ab initio calculations using the stress-strain method. The electronic structure of hcp PdH_2_ is also analyzed by band structure and DOS projected on atoms and orbitals. From the obtained results, the mechanical and electronic properties of the PdH_2_ are analyzed.

## Results and Discussion

### Crystal structure

**Figure 1:**
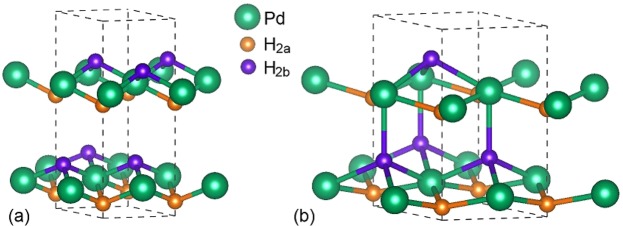
The hcp structure of PdH_2_ at (**a**) 0 GPa and (**b**) 20 GPa. The Pd atoms occupy 2b site in green color, H atoms occupy two different Wyckoff positions, one is 2a in yellow color and the other is 2b in purple color.

A global energy minimum phase of PdH_2_ structure was found by ab initio random structure searching technique as shown in Fig. 1. It is an hcp structure with the space group P6_3_mc. In this structure, Pd atoms are positioned at 2b (1/3 2/3 z) and H atoms occupy the 2a (0 0 z) and 2b (1/3 2/3 z) site. The atomic symmetry of both Pd and H atoms is 3m. As shown in Fig. 1(a), at 0 GPa, Both of H atoms 2a sites (H_2*a*_) and 2b sites (H_2*b*_) are three-coordinated with Pd atoms. As the pressure increases above 15 GPa (see in Fig. 1(b)), the coordination number of H_2*b*_ atoms with Pd atoms increased to four.

**Figure 2:**
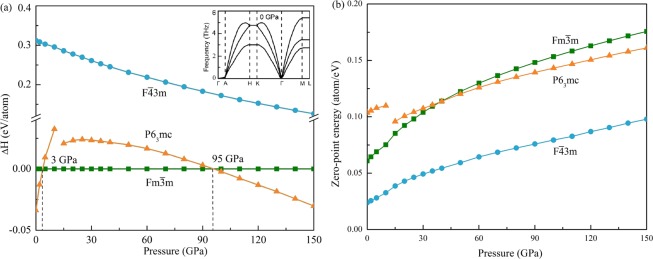
The enthalpy differences and zero-point energy as a function of pressure (**a**) ΔH for hcp and F$\overline{4}$3m PdH_2_ relative to Fm$\overline{3}$m PdH_2_; (**b**) zero-point energy.

For comparison, the fcc PdH_2_ with the space group Fm$\overline{3}$m and F$\overline{4}$3m^21^ were considered. In the Fm$\overline{3}$m structure, Pd and H atoms are located at the Wyckoff positions 4a (0 0 0) and 8c (1/4 1/4 1/4). The atomic symmetries of Pd and H atoms are $\overline{4}$3m and m3m, respectively. The 8c H (H_8*c*_) atoms occupy two tetrahedral (T) sites. In the F$\overline{4}$3m structure, Pd atoms located at the Wyckoff positions 4a (0 0 0) and the H atoms occupy the 4b (1/2 1/2 1/2) and 4c (1/4 1/4 1/4) site. The atomic symmetries of Pd and H atoms are $\overline{4}$3m. The 4b H (H_4*b*_) atoms and 4c H (H_4*c*_) atoms occupy octahedral (O) sites and T sites, respectively. The enthalpy differences $\varDelta$H for P6_3_mc and F$\overline{4}$3m PdH_2_ relative to Fm$\overline{3}$m PdH_2_ are obtained. Figure 2 shows the enthalpy difference $\varDelta$H including zero-point energy and zero-point energy as a function of pressure. The ΔH takes positive values for F$\overline{4}$3m structure at all studied pressure indicating that the F$\overline{4}$3m structure is unstable than the Fm$\overline{3}$m structure. At the pressure lower than 3 GPa or higher than 95 GPa, ΔH of P6_3_mc structure takes negative values. It suggests that the hcp structure is more stable than the F$\overline{4}$3m structure in these pressure ranges. Phonon dispersion in the whole Brillouin zone of hcp PdH_2_ at 0 GPa is shown in Fig. 2(a). The inexistence of imaginary frequencies indicates the dynamic stability of the phases at 0 GPa.

The zero-point energy (ZPE) of the system is defined as the free energy of the system at 0 K. To improve accuracy, the zero point vibration energy was corrected in the energy calculations. The zero-point energy monotonous increase with the increase of pressure, except for the P6_3_mc structure at 15 GPa as shown in Fig. 2(b). The reason for the discontinuity of zero energy is that the hcp PdH_2_ undergoes an isostructural phase transition when the pressure is about 15 GPa. The detailed discussion is carried out in the later discussion on structural parameters. The results show that the P6_3_mc PdH_2_ is, in fact, a different phase under pressure. For comparison, we have included the hcp P6_3_mc and fcc Fm$\overline{3}$m structures in the subsequent calculations of the structural parameters.

**Figure 3:**
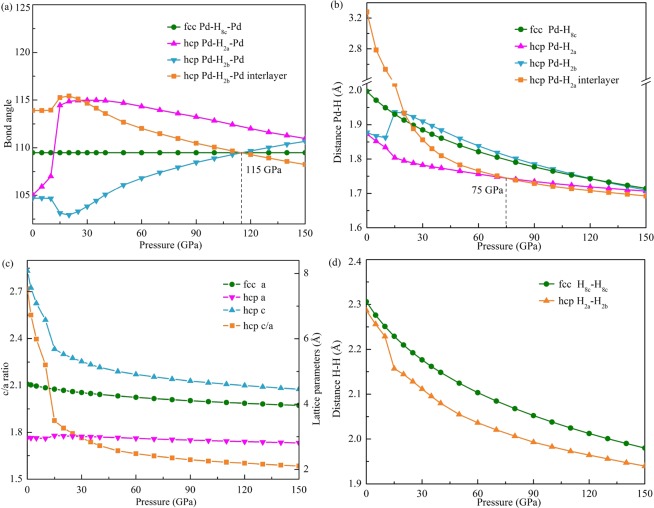
Structural parameters of PdH_2_ as a function of pressure: (**a**) bond angle of Pd-H-Pd; (**b**) distance between Pd and H atoms; (**c**) c/a ratio of hcp structure and lattice parameter a and c; (**d**) distance between two H atoms.

The structural parameters of PdH_2_ can be obtained by full relaxation of the structure. Figure 3 shows the structural parameters as a function of pressure.

The bond angle of Pd-H-Pd as a function of pressure is shown in Fig. 3(a). at 0 GPa, the inlayer angles Pd-H_2*a*_-Pd and Pd-H_2*b*_-Pd of hcp structure are smaller than the angle Pd-H_8*c*_-Pd of fcc structure, the interlayer angle Pd-H_2*b*_-Pd is bigger than the Pd-H_8*c*_-Pd. When the pressure is less than 15 GPa, the inlayer and the interlayer Pd-H_2*b*_-Pd angles have a slight change with the increase of pressure. All the Pd-H-Pd bond angles changed significantly at 15 GPa due to the influence of the interlayer bonding force. The Pd-H_2*a*_-Pd has a sharp increase at 15 GPa which makes it bigger than the Pd-H_8*c*_-Pd. The hcp lattice parameter a has a slight increase at 15 GPa. As the PdH_2_ is further compressed, the inlayer Pd-H_2*a*_-Pd and interlayer Pd-H_2*b*_-Pd decrease and inlayer Pd-H_2*b*_-Pd increases. The H_2*b*_ atoms occupy the ideal T sites at 115 GPa.

Figure 3(b) shows the distance between Pd and H as a function of pressure. The Pd-H distance decreases with the increase of pressure. The Pd-H_2*a*_ distance and Pd-H_2*b*_ distance are equal to 1.87 Å and less than the Pd-H_8*c*_ distance which is 2.00 Å at 0 GPa. When the pressure is lower than 15 GPa, the Pd-H_2*a*_ and Pd-H_2*b*_ are similar in size. In addition, Pd-H_2*a*_ and Pd-H_2*b*_ are still less than the Pd-H_8*c*_. Pd-H_2*a*_ is smaller to the Pd-H_2*b*_ and the difference for Pd-H_2*b*_ relative to Pd-H_2*a*_ increases with the increase of pressure. When the pressure exceeds 15 GPa, the Pd-H_2*b*_ is similar to the Pd-H_8*c*_ in size. The Pd-H_2*b*_ is larger than the Pd-H_2*a*_ and the difference for Pd-H_2*b*_ relative to Pd-H_2*a*_ decreases with the increase of pressure. The interlayer Pd-H_2*b*_ distance is almost two times bigger than Pd-H_2*a*_ and Pd-H_2*b*_ at 0 GPa. The interlayer bonding force between Pd and H is very weak. When the external pressure is applied, the c-axis is rapidly compressed. The interlayer bonding force between Pd and H increases with the increase of pressure. The interlayer Pd-H_2*b*_ is similar to other Pd-H distance in size when the pressure exceeds 15 GPa. The interlayer Pd-H_2*b*_ and Pd-H_2*a*_ are equal at 75 GPa, and the bond length does not change with the increase of pressure. The interlayer bonding force between Pd and H increased significantly at around 15 GPa. All of the Pd-H distance tends to 1.7 Å as the pressure up to 150 GPa.

As is shown in Fig. 3(c), the c/a ratio decreases dramatically from 2.718 to 1.875 with the increase of pressure from 0 GPa to 15 GPa. As the PdH_2_ is further compressed, the decrease of c/a ratio becomes slower with the increase of pressure. This indicates that the interlayer bonding force between Pd and H atoms is significantly enhanced when the pressure reaches above 15 GPa. The c/a ratio almost tends to an ideal value of 1.613 as the pressure exceeds 100 GPa. The c/a ratio changes with the pressure, indicating that the hcp PdH_2_ is highly anisotropic. The lattice parameter a of both hcp and fcc PdH_2_ decreases with the increase of pressure, except a slight increase at around 15 GPa. When pressure is lower than 15 GPa, the c decreases rapidly compare to a with the increase of pressure. With further increase in pressure, the decrease of c becomes slower.

Pressure affects the distance between the two H atoms. As is shown in Fig. 3(d), the H-H distance decreases with the increase of pressure. The hcp H_2*a*_-H_2*b*_ distance is always less than fcc H_8*c*_-H_8*c*_ distance. The H_2*a*_-H_2*b*_ distance is closer to H_8*c*_-H_8*c*_ distance at a pressure below 15 GPa than the pressure above 15 GPa.

Discontinuous change of bond length, bond angle, c/a, lattice parameter a, and the crystal symmetry remains unchanged at 15 GPa, indicating that there is an isostructural phase transition of hcp PdH_2_. For hcp PdH_2_, a = 2.973 Å and c/a = 2.718 at 0 GPa and a = 3.034 Å and c/a = 1.875 at 15 GPa.

### Mechanical properties

Elastic constants are the quantities to characterize the elasticity of materials which determine the response of materials to external forces. Elastic constants C_*ij*_ of hcp PdH_2_ as a function of pressure are listed in Table 1. It shows that all of the C_*ij*_ increases monotonously with the increase of pressure. The magnitude of all the C_11_, C_12_, C_13_ and C_33_ are greater than the magnitude of the applied pressure except for the C_44_. The smaller increasing rate in the value of C_44_ leads to that the shear deformation increase faster than before with the increase of pressure. It implies that the pressure reduces the stability of the structure.

**Table 1:** Elastic constants C_*ij*_ (GPa) of PdH_2_ as function of pressure P (GPa).

P	C_11_	C_12_	C_13_	C_33_	C_44_
0	154.8	54.1	14.2	21.0	-3.8
5	187.3	73.0	42.9	46.1	-2.2
10	213.4	92.0	66.3	61.8	0.9
15	229.5	148.5	111.3	116.0	20.2
20	248.2	162.8	128.7	148.1	24.2
50	354.0	241.9	202.5	250.7	37.2
100	483.4	361.0	302.7	442.3	46.8
150	588.2	482.3	427.3	558.3	58.1

The obtained bulk modulus B, shear modulus G, B/G ratio, Young’s modulus E and Poisson’s ratio *ν* of PdH_2_ as a function of pressure are listed in Table 2. The B, G and E increase with the increase of pressure. All the B/G values are larger than 1.75 showing a ductile character that does not change with external pressure. The *ν* varies from 0.40 to 0.44. All of the *ν* is larger than 0.25, indicating that the PdH_2_ is an ionic bonding material.

**Table 2:** The calculated bulk modulus B (in GPa), shear modulus G (in GPa), B/G, Young’s modulus E (in GPa) and Poison’s ratio *ν* of PdH_2_ as a function of pressure P (in GPa).

P	B	G	B/G	Y	*ν*
0	37.8	6.4	5.9	18.2	0.42
5	64.0	11.0	5.8	31.1	0.42
10	82.9	16.1	5.2	45.3	0.41
15	131.0	27.7	4.7	77.7	0.40
20	154.6	31.7	4.9	89.1	0.40
50	242.4	45.8	5.3	129.3	0.41
100	369.2	58.9	6.3	167.9	0.42
150	488.2	59.6	8.2	171.9	0.44

**Figure 4:**
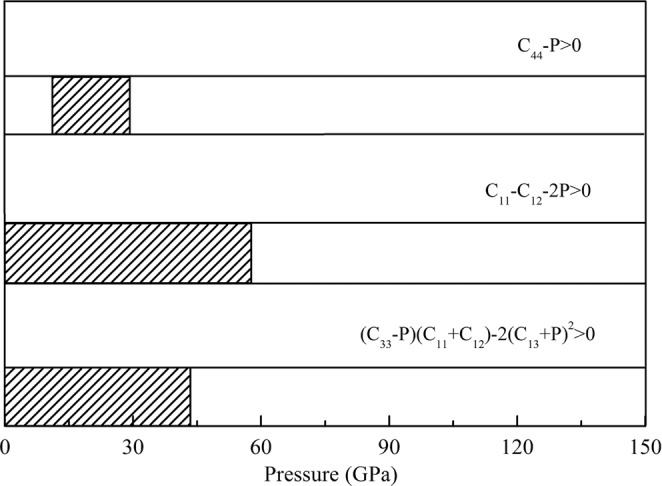
The mechanical stability criteria of hcp PdH_2_. The fill parts indicate that the stability condition is satisfied.

The mechanical stability for hcp PdH_2_ as shown in Fig. 4. When the pressure range is 13 to 29 GPa, the C_44_ − P > 0 is fulfilled. It means that the shear strain in (100) plane does not cause mechanical instability under the corresponding pressure range. The C_11_ − |C_12_| − 2P convert to negative when pressure reaches around 57 GPa. For hcp structure, the shear elastic constants C_66_ $=$ C_11_ − C_12_, hence the shear strain in (001) plane does not cause mechanical instability when the pressure is below 57 GPa. The C_11_ − P > 0 and C_33_ − P > 0 are fulfilled (see Table 1), indicating that one axial strain along the [100] and [001] directions does not lead to mechanical instability. The coupling strain along the [100] and [120] directions will lead to mechanical instability because of the C_11_ − |C_12_| − 2P < 0 when the pressure is above 57 GPa. The (C_33_ − P) (C_11_ + C_12_) − 2(C_13_ + P)^2^ > 0 is fulfilled when the pressure lower than 43 GPa. It indicates that the coupling strain along the [100], [120] and [001] directions does not cause mechanical instability when the pressure is in this pressure range. All the mechanical stability criteria are all fulfilled in the pressure range of 13 to 29 GPa. While the enthalpy differences for hcp PdH_2_ relative to fcc PdH_2_ are positive at this pressure range which implies that the hcp PdH_2_ can be a metastable structure in the pressure range of 13 to 29 GPa. It means that the hcp PdH_2_ is metastable when the c/a is in the range of 1.765 to 1.875. When the pressure is lower than 13 GPa or higher than 29 GPa, the slip of PdH_2_ will occur between the {001} plane.

The projection of Pd-H_2*a*_ and Pd-H_2*b*_ bond in the {001} plane is along the [120] and [1$\bar{1}$0] direction. The strain along the [120] and [1$\bar{1}$0] direction is related to C_11_ and C_11_ − C_12_. Therefore, the Pd-H_2*b*_ bond is the major factor that limits the mechanical stability of hcp PdH_2_.

Elastic anisotropic is also a fundamental parameter regarding mechanical properties. In this work, the c/a value changes with pressure. It means that the structure is always varying with the applied pressure. When the c/a value is in 1.765–1.875, the PdH_2_ is mechanically stable. Here, we have discussed the anisotropy parameters of PdH_2_ with c/a 1.827. For an isotropic medium, ΔP = ΔS_1_ = ΔS_2_ = 1. PdH_2_ is anisotropic. For PdH_2_, the compressional anisotropy ΔP = 0.60. The PdH_2_ is more easily compressed in the [001] direction than the [100] direction. The shear anisotropy ΔS_1_ and ΔS_2_ are 1.43 and 0.57, respectively. Due to the small C_44_, the shear anisotropy is large. It indicates that the largest shear deformation occurs in {100} plane and the slip is most likely to occur between planes parallel to {001} plane. All the anisotropy parameters are far away from 1(one), which means that the hcp PdH_2_ is highly anisotropic. The results indicate that the Pd-H bonds are stronger in the layer which is parallel to the {001} plane than between the layers.

### Electronic structure

**Figure 5:**
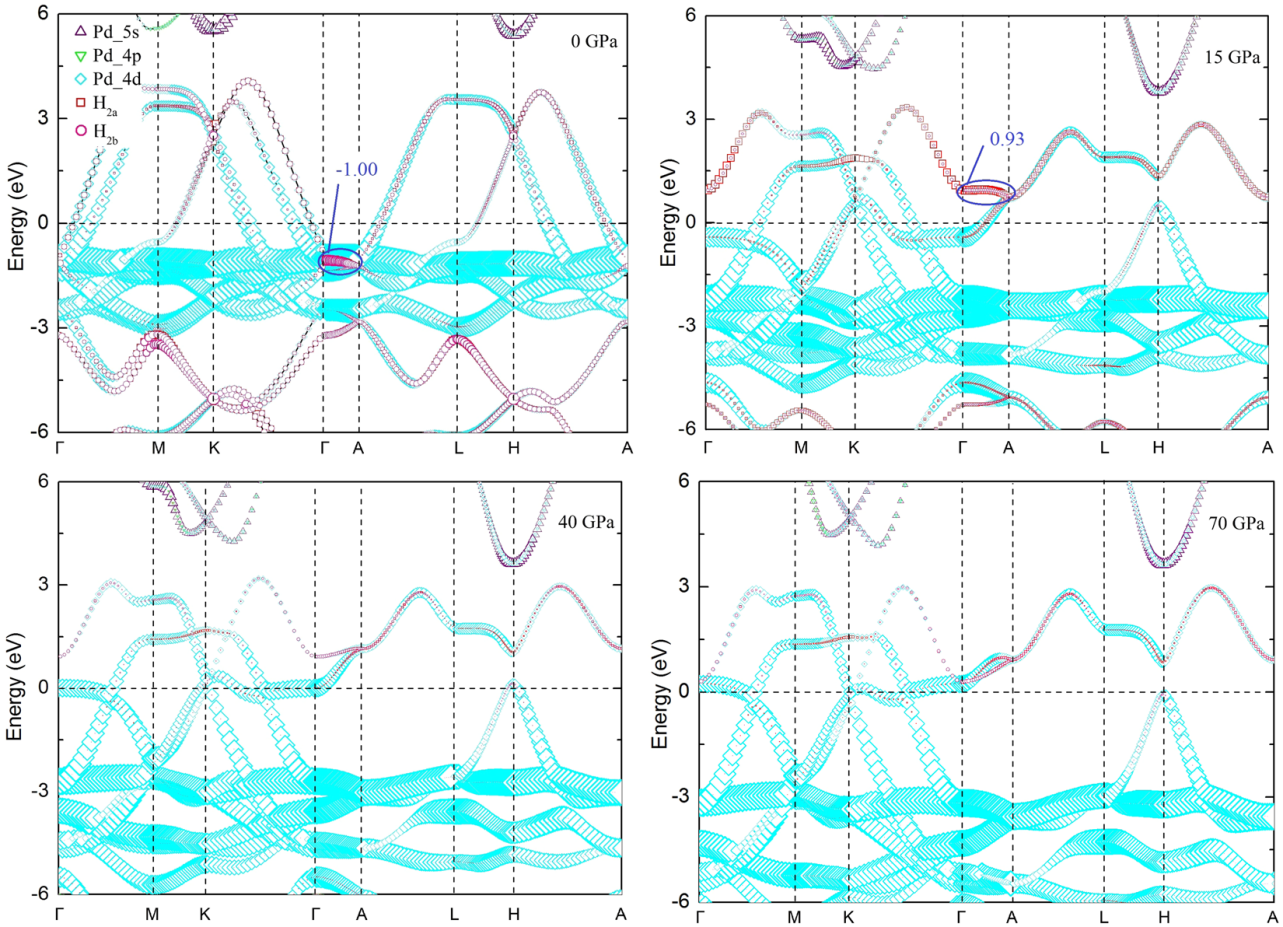
Electronic fat band for PdH_2_ at the different pressure. The Fermi level is set to be at zero. The size of the symbol represents the weight of projection on orbitals.

The fat band along the high-symmetry directions of the BZ for PdH_2_ at different pressure are shown in Fig. 5. The d-band on Pd atom has the main contribution to the electronic structure. The flat bands near the Fermi level suggest that the hcp PdH_2_ is potential superconducting material. The band moves to lower energy with the increase of pressure. It also shows that two type-I Dirac point appears at K and H high-symmetry points indicating that the hcp PdH_2_ is a topological-like structure. When the pressure increase from 0 GPa to 15 GPa, the gap between Dirc point appears. As the pressure increases from 0 GPa to 15 GPa, the flat band of H_2*b*_ and H_2*a*_ along Γ to A point move from −1.00 to 0.93. The anti-bonding was formed at 15 GPa. This sudden change in electronic structure at 15 GPa is due to the electronic topological transition of Fermi surface morphology. There are flat bands along with the high-symmetry directions [100] and [110] in the BZ. With the increase of pressure, the flat bands move to high energy because the partially occupied Pd 4d states were excited to a higher energy state. The flat bands move to the Fermi level at 40 GPa. When the pressure is higher than 40 GPa, the flat bands move across the Fermi level.

**Figure 6:**
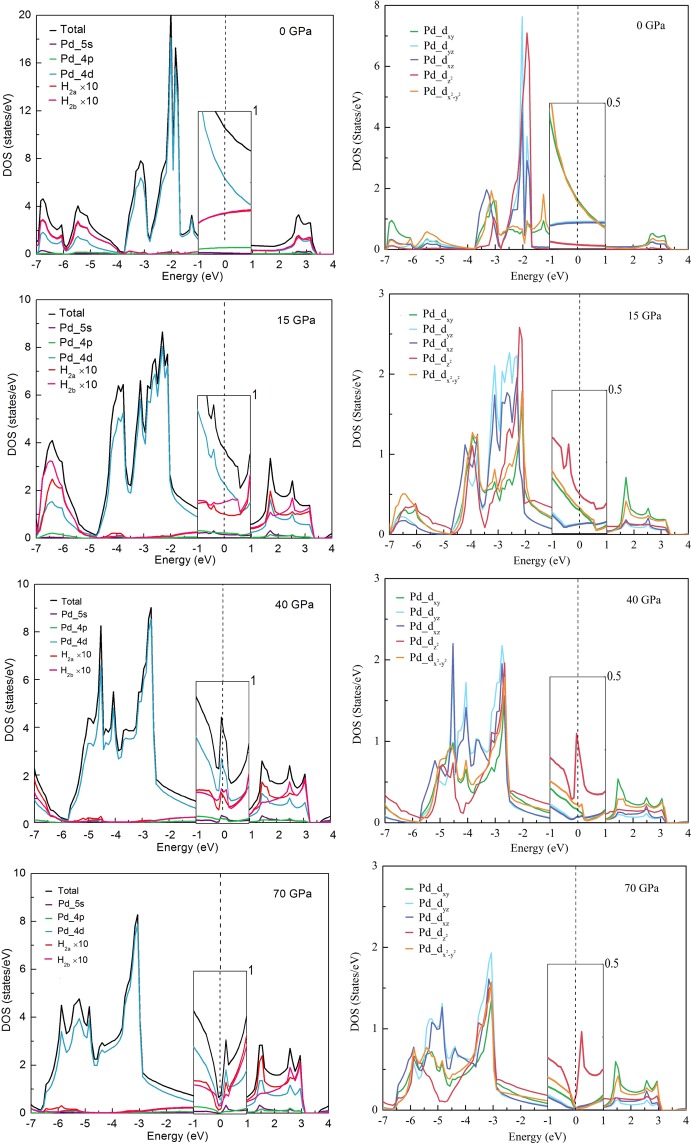
DOS projected on atoms and orbitals for PdH_2_ at the different pressure. The Fermi level is set to be at zero.

The density of states (DOS) projected on atoms and orbitals for PdH_2_ at different pressure is shown in Fig. 6. This figure shows a pressure-induced metal to semimetal transition in the PdH_2_. It is seen that the Pd 4d states have the main contribution to the total DOS at around Fermi level. The H_2*a*_ 1s and H_2*b*_ 1s states have a similar contribution to the DOS at around the Fermi level at 0 GPa, as shown in Fig. 6 (0 GPa).

Applied pressure reduces the distance between two layers of PdH_2_. As is shown in Fig. 6 (15 GPa), the interaction between Pd and H due to the two peaks at around the Fermi level. The Pd ${\text{d}}_{{z}^{2}}$ states produce the main contribution to the DOS at around Fermi level. Above the Fermi level, the first peak shows that the H_2*b*_ 1s states contribute more to DOS than the H_2*a*_ 1s states. The first peak below the Fermi level shows that the H_2*a*_ 1s and H_2*b*_ 1s states almost have the same contribution to the DOS. The Pd ${\text{d}}_{{z}^{2}}$ states interact with the H 1s states lead to an increase in interlayer bonding force between Pd and H.

The peaks around the Fermi level move towards the Fermi level as the increase of pressure. As can be seen in Fig. 6 (40 GPa), the peak is on the Fermi level. This peak is derived from the flat zone along with the high-symmetry directions [100] and [110] in the Brillouin zone. It suggests that the hardness of the material is strengthened.

As the PdH_2_ is further compressed, as in Fig. 6 (70 GPa), the peak moves above the Fermi level. The DOS has a minimum value at the Fermi level. The results show a phase transition from metal to semimetal in the PdH_2_ under pressure.

The DOS of both H_2*a*_ and H_2*b*_ 1s states are flat at around Fermi level at 0 GPa. However, they are not flat as the PdH_2_ is compressed. When the pressure was applied, some small peaks for H 1s states can be found at around Fermi level because of the Pd 4d states interact with other states. Hybridization of Pd ${\text{d}}_{{z}^{2}}$ states and H 1s states increases the bonding force between layers of PdH_2_. These results indicate that the PdH_2_ is more stable under pressure than at 0 GPa.

## Conclusions

In conclusion, a palladium hydride (PdH_2_) with high hydrogen concentration is reported. The mechanical and electronic properties of PdH_2_ were investigated by the ab initio calculations. We have reported elastic parameters such as bulk modulus, shear modulus, Young’s modulus, Poisson’s ratio within Hill approximation. It has been found that the hcp PdH_2_ is mechanically stable when the c/a ratio is between 1.765 to 1.875. The Pd-H_2*b*_ bond is the major factor that limits the mechanical stability of this structure. The analysis of mechanical stability and anisotropy also shows that the interatomic forces of hcp PdH_2_ are weaker between the layers which is parallel to the {001} plane than in the layer. Therefore, the cause of PdH_2_ structural instability is the slip between {001} planes. Our study shows that the PdH_2_ is dynamic stable at 0 GPa and the interatomic forces between layers increases with the increase of pressure. The results also show that there is an electronic topology transition of hcp PdH_2_ at 15GPa. Our results suggest that PdH_2_ can be stabilized in a metastable form.

### Methods

The structural and elastic constants of PdH_2_ have been studied by performing first principles calculations. The calculations were achieved based on the density functional theory (DFT)^23^. The ab initio random structure searching technique^24^ was used to find potential PdH_2_ crystal structures at ambient and high pressure. The random search study was performed at 0, 50, and 100 GPa, with 1, 2, 3, and 4 PdH_2_ units per simulation cell. The ab initio random structure searching technique generated unit cells of random shapes with reasonable volumes by calculating the ground state structure as well as determining the positions of PdH_2_ formula in the cells. A plan-wave basis-set energy cutoff of 260 eV and an initial Brillouin Zone (BZ) sampling grid of 2*π* × 0.07 Å^−1^ were found to be sufficient for the initial searches. The generalized gradient approximation (GGA) with the Perdew-Burke-Ernzerhof (PBE)^25^ parameterization for the exchange-correlation functional and ultrasoft pseudopotential^26^ were used for the structure searches by CASTEP code^27^.

The stable structure of PdH_2_ was studied by ab initio lattice dynamics with a supercell approach, as implemented in the Vienna ab initio Simulations Package (VASP) code^28^ and the phonopy package^29^. The structure was done using the projector augument-wave (PAW)^30^ method and GGA was used to describe the electronic exchange-correlation effects. The calculation used 2 × 2 × 2 supercells, with consisting of 48 atoms, for P6_3_mc PdH_2_ by using a plane-wave basis-set energy cutoff of 700 eV and sampling the BZ with fixed k-mesh 13 × 13 × 4.

The geometry relaxation and elastic constants calculations were performed using the VASP code. The exchange-correlation functional was described within the GGA of PBE. The outer electron configuration is 4p^6^5s^1^4d^9^ for Pd. The plane-waves kinetic energy cut-off was set to be 900 eV. The “High” precision setting was used to avoid wrap around errors in the calculations. The vdW-DF2^31^ functional was used to include the van der Waals interactions in the PBE functional. In a weakly bonded layered system, this function can calculate accurately equilibrium spacing and binding energy compare to the vdW-DF function^32^. The van der Waals (vdW) forces include attraction and repulsions between atoms, molecules, and surfaces, as well as other intermolecular forces. They differ from covalent and ionic bonding in that they are caused by correlations in the fluctuating polarizations of nearby particles. For the sparse systems, including soft matter, van der Waals complexes, and layered materials, which have interparticle separations, the vdW forces are important for the interactions of nonlocal and long-ranged.

For the structural optimization and self-consistent calculations, the energy and forces convergence criterion of the electronic self-consistency were chosen as 10^−8^ eV and 10^−3^ eV/Å per atom. The k-meshes were generated automatically to divide the BZ in each direction. A k-mesh 17 × 17 × 17 was generated by Gamma method for hcp PdH_2_ and a 22 × 22 × 22 was generated by Monkhorst-Pack method for fcc PdH_2_. These settings ensure a high convergence of 1 meV per unit cell in the total energy and accurate values of the forces in the atoms. For calculations of the hcp PdH_2_ electronic band and DOS, the k-mesh was increased to 27 × 27 × 27.

Ab initio calculations were performed for enthalpies as a function of pressure. The enthalpy H at 0 K can be obtained by using the expression:

1 $$H={E}_{0}+{E}_{ZPE}+PV$$

where E_0_ is the total energy, E_*ZPE*_ is the zero-point energy, P is the hydrostatic pressure and V is the volume.

For the hcp PdH_2_, the elastic constants were calculated by using VASP software based on the strain-stress approach^33^. The number of k-mesh was increased to 27 × 27 × 27 for elastic constants calculations. A 0.01 Å positive and negative displacement was applied for each atom. For an hcp crystal, there are five independent elastic constants, C_11_, C_12_, C_13_, C_33_ and C_44_. According to Hooke’s law, the stress-strain relationship can be written as:

2 $$(\begin{array}{c}{\sigma }_{1}\\ {\sigma }_{2}\\ {\sigma }_{3}\\ {\tau }_{1}\\ {\tau }_{2}\\ {\tau }_{3}\end{array})=(\begin{array}{cccccc}{C}_{11} & {C}_{12} & {C}_{13} & 0 & 0 & 0\\ {C}_{12} & {C}_{11} & {C}_{13} & 0 & 0 & 0\\ {C}_{13} & {C}_{13} & {C}_{33} & 0 & 0 & 0\\ 0 & 0 & 0 & {C}_{44} & 0 & 0\\ 0 & 0 & 0 & 0 & {C}_{44} & 0\\ 0 & 0 & 0 & 0 & 0 & {C}_{66}\end{array})(\begin{array}{c}{\varepsilon }_{1}\\ {\varepsilon }_{3}\\ {\varepsilon }_{3}\\ {\gamma }_{1}\\ {\gamma }_{2}\\ {\gamma }_{3}\end{array})$$

where C_66_ = 1/2 (${\text{C}}_{11}-{\text{C}}_{12}$). The elastic tensor is determined by performing six finite distortions of the lattice and deriving the elastic constants from the strain-stress relation.

The bulk modulus and shear modulus are used to describe the material’s response to compress and shear stress. Here, we use the Hill approximations^34^, which are an average of the Voigt^35^ and Reuss^36^ elastic constants, to calculate the bulk modulus B and shear modulus G. The Voigt and Reuss approximations, labeled with subscripts V and G, are defined by:

3 $$\begin{array}{rcl}{B}_{R} & = & \frac{({C}_{11}+{C}_{12}){C}_{33}-2{C}_{13}^{2}}{{C}_{11}+{C}_{12}+2{C}_{33}-4{C}_{13}}\\ {B}_{V} & = & \frac{1}{9}(2{C}_{11}+2{C}_{12}+4{C}_{13}+{C}_{33})\\ {G}_{R} & = & \frac{15}{4(2{S}_{11}+{S}_{33}-2{S}_{13}-{S}_{12})+3(2{S}_{44}+{S}_{66})}\\ {G}_{V} & = & \frac{1}{30}({C}_{11}+{C}_{12}+2{C}_{33}-4{C}_{13}+12{C}_{44}+12{C}_{66})\end{array}$$

where S_*ij*_ represents the elements of elastic compliance matrix which is equal to the reciprocal of elastic constant matrix. Then, the bulk modulus B and shear modulus G were obtained by:

4 $$\begin{array}{rcl}B & = & 0.5({B}_{V}+{B}_{R})\\ G & = & 0.5({G}_{V}+{G}_{R})\end{array}$$

The G represents the resistance to plastic deformation and the B represents the resistance to fracture. The value of B/G is used to characterize the ductility of the material^37^. The material behaves in a ductile nature if B/G > 1.75, otherwise a brittle nature.

Young’s modulus Y describes the response to linear stress. Poisson’s ratio $\nu$ measures the phenomenon of deformation perpendicular to the loading direction when the material is compressed or stretched. The bond sorting can use the value of the $\nu$. The $\nu$ is very small than 0.25 for a covalently bonded compound. While, for a typical ionic compound, the $\nu$ is nearly 0.25 or more. Young’s modulus and Poisson’s ratio^38^ were calculated with the expressions:

5 $$\begin{array}{rcl}Y & = & 9B/(3B/G+1)\\ \nu  & = & (3B/G-2)/(6B/G+2)\end{array}$$

A crystalline structure is mechanical stable, if elastic energy is always positive. Elastic constants are used to determine the mechanical stability. At 0 GPa, the mechanical stability criteria for hcp structure are featured as^39^:

6 $$\begin{array}{c}{C}_{44} > 0\\ {C}_{11}-|{C}_{12}| > 0\\ {C}_{33}({C}_{11}+{C}_{12})-2{C}_{13}^{2} > 0\end{array}$$

At the compression, the mechanic stability criteria are pressure related^40^:

7 $$\begin{array}{c}{C}_{44}-P > 0\\ {C}_{11}-|{C}_{12}|-2P > 0\\ ({C}_{33}-P)({C}_{11}+{C}_{12})-2{({C}_{13}+P)}^{2} > 0\end{array}$$

where P is external pressure.

The mechanical stability criteria show the response of the material to axial and tangential strain. The C_44_ − P > 0 is related to shear strain in (100) face. The C_11_ − C_12_ − 2P $\equiv$ C_66_ − P > 0 is related to shear strain in (001) face. The C_11_ − P > 0 and C_33_ − P > 0 are related to the axial strain along the [100] and [001] direction, respectively. The C_11_ − |C_12_| − 2P > 0 is related to the coupling strain along the [100] and [120] directions, which is same as the strain along the [1$\bar{1}$0] direction. The (C_33_ − P) (C_11_ − P) − (C_13_ + P)^2^ > 0 is related to the coupling strain along the [100] and [001] directions. The (C_33_ − P) (C_11_ + C_12_) − 2(C_13_ + P)^2^ > 0 is related to the coupling strain along the [100], [120] and [001] directions.

Elastic anisotropic is a fundamental parameter for mechanical properties. For an hcp crystal, the elastic anisotropic is described by the following formulas^41^:

8 $$\begin{array}{rcl}{\Delta }_{P} & = & {C}_{33}/{C}_{11}\\ {\Delta }_{S1} & = & ({C}_{11}+{C}_{33}-2{C}_{13})/4{C}_{44}\\ {\Delta }_{S2} & = & 2{C}_{44}/({C}_{11}-{C}_{12})\end{array}$$

where ΔP is anisotropy for compressional wave, ΔS_1_ and ΔS_2_ are anisotropy for shear wave, polarized perpendicular to the basal plane and polarized in the basal plane, respectively. These three parameters characterize the anisotropy of the three main acoustic modes. The acoustic anisotropy in turn indicates the anisotropy of the elastic constants.
